# Genetic evidence of *Coxiella burnetii* infection in acute febrile illnesses in Iran

**DOI:** 10.1371/journal.pntd.0007181

**Published:** 2019-02-11

**Authors:** Saber Esmaeili, Ashraf Mohabati Mobarez, Mohammad Khalili, Ehsan Mostafavi, Pardis Moradnejad

**Affiliations:** 1 Department of Bacteriology, Faculty of Medical Sciences, Tarbiat Modarres University, Tehran, Iran; 2 Department of Pathobiology, Faculty of Veterinary Medicine, Shahid Bahonar University of Kerman, Kerman, Iran; 3 Department of Epidemiology and Biostatics, Research Centre for Emerging and Reemerging Infectious Diseases, Pasteur Institute of Iran, Tehran, Iran; 4 National Reference Laboratory for Plague, Tularemia and Q Fever, Research Centre for Emerging and Reemerging Infectious Diseases, Pasteur Institute of Iran, Akanlu, Kabudar-Ahang, Hamadan, Iran; 5 Rajaie Cardiovascular Medical and Research Center, Iran University of Medical Sciences, Tehran, Iran; Institut Pasteur, FRANCE

## Abstract

Mounting evidence suggests that Q-fever is more prevalent in Iran than originally believed. However, in most parts of the country, clinicians do not pay enough attention to Q fever in their differential diagnosis. The aim of this study was to investigate the prevalence of *Coxiella burnetii* in suspected cases of acute Q fever in north-western Iran using molecular techniques. Febrile patients were enrolled in the study and investigated for *C*. *burnetii* infection. Sera samples were tested using real-time PCR for detection of *IS1111* gene, and positive samples were confirmed with nested PCR. Nine patients (4.2%) out of 216 suspected cases were positive for *C*. *burnetii*. Weakness and fatigue, headache, and lethargy were the most prevalent clinical symptoms in acute Q fever patients. According to the results of this study and other reports of human cases in Iran, the diagnosis system of Q fever in Iran should be urgently revamped.

## Author summary

Q fever is a zoonotic infectious disease caused by *Coxiella burnetii*. Domestic ruminants are the most common source of human infection. Main route of transmission to humans is inhalation of infected aerosols and dust with *C*. *burnetii*. Acute Q fever is usually presented as a non-specific febrile and self-limiting influenza-like illness, but in severe acute cases, may manifest as atypical pneumonia or hepatitis. In Iran, Q fever is an endemic disease with high seroprevalence among humans and domestic animals. However, human Q fever cases remain undiagnosed in most regions of Iran, especially because most clinicians fail to spot this disease in their differential diagnosis. The aim of this study was to investigate the prevalence of acute Q fever in suspected cases (216 suspected cases) using molecular techniques. Nine acute Q fever patients were diagnosed by Real-time PCR and Nested PCR. Weakness and fatigue, headache, and lethargy were the most prevalent clinical symptoms in positive cases. Human Q fever cases described in this, and previous studies, indicate the need to implement diagnostic techniques for this disease across the country.

## Introduction

Q fever is a zoonotic infectious disease caused by *Coxiella burnetii*, an obligate intracellular bacterium. Outbreaks have been reported in both developing and developed countries. Domestic ruminants (cattle, sheep, and goats) are the most common source of human infection [1]. In animals, *C*. *burnetii* infection is mostly asymptomatic, but can lead to abortion, stillbirth, infertility, endometritis and metritis. Infected animals shed *C*. *burnetii* in urine, feces, milk, and especially in birth or abortion products. Main route of transmission to humans is inhalation of infected aerosols and dust with *C*. *burnetii*. Ingestion of contaminated raw milk and dairy products, skin or mucosal contact, tick bites, blood transfusion, sexual transmission, and embryo transfer are less common routes of infection transmission to humans [1, 2].

In humans, the incubation period for primary infection has been estimated to be between 7 and 32 days after exposure [3]. Acute Q fever is the primary form of infection by *C*. *burnetii*, and more than half of the patients are asymptomatic. Acute Q fever usually presents itself as a non-specific febrile self-limiting influenza-like illness, but may also manifest as atypical pneumonia or hepatitis [4]. Cardiac involvement, neurological signs, acute lymphadenitis, cholecystitis, autoimmunity, bone marrow involvement, and dermatological signs have also been reported in some acute cases of Q fever [1]. The main manifestation of chronic Q fever is life-threatening endocarditis and vascular infection. Other less common forms of chronic Q fever includes abortion, lymphadenitis, osteomyelitis, prosthetic joint arthritis and osteoarticular infection [1, 5].

Due to the wide range of clinical symptoms of Q fever in humans, clinical diagnosis of the disease can be challenging based on symptoms alone. Therefore, laboratory confirmation is a major and crucial part in the diagnosis of clinical cases [4]. Laboratory diagnosis of Q fever in humans is mainly based on serological tests, ELISA and IFA as the gold standard test. *C*. *burnetii* isolation from clinical samples is not performed in most diagnostic laboratories because it requires eukaryotic cell cultures and access to BSL3 facilities. In recent years, PCR-based molecular assays were developed to detect *C*. *burnetii* in clinical specimens. PCR-based techniques are more adapted than serology for early diagnosis of acute Q fever because of delay in the antibody response, which is detectable only after 2–3 weeks following infection [1, 6].

In Iran, Q fever is an endemic disease with high seroprevalence among humans and domestic animals [7]. In recent years, many acute and chronic Q fever cases have been reported in Iran [8–11]. Furthermore, several investigations have been published on the prevalence of Q fever among domestic livestock in Iran [7]. However, human cases of Q fever remain undiagnosed in most regions of Iran, especially because most clinicians do not consider this disease in their differential diagnosis.

The incidence of acute Q fever is underestimated in most parts of the world. The clinical presentations in acute Q fever patients is very pleomorphic, nonspecific and confusing. Less than 4% of patients with acute fever require hospitalization [1, 12]. This disease is often disregarded by physicians and healthcare system and diagnosis relies upon the physicians’ awareness of the clinical symptoms of acute Q fever and access to reliable diagnostic laboratory facilities including serology and PCR [4]. Diagnosed acute cases with *C*. *burnetii* must be treated promptly to avoid to chronic Q fever [13]. The rapid and timely diagnosis of acute fever can help cure patients and avoid the spreading of the disease. Conducting molecular studies, such as the current study, can help to rapidly diagnosis of patients with acute febrile illness, as well as can raise awareness and sensitivity of clinicians and the health care system about Q fever in Iran. The aim of this study was to investigate the prevalence of *C*. *burnetii* in suspected cases of acute Q fever by molecular methods.

## Material and methods

### Study design

The samples of this study were collected from two surveys carried out in Tabriz County in the East Azerbaijan Province (North West of Iran) in 2013 and Ghaemshahr County in the Mazandaran province (Northern Iran) in 2015–2016.

### Sampling

Patients which met the following criteria, were enrolled to the study as suspected acute Q fever cases:

- Clinical symptoms: Febrile patients (acute undifferentiated febrile illnesses) had acute lower respiratory infection (atypical pneumonia) with at least two other symptoms including chills, headache, fatigue, shortness of breath, and myalgia, for which the causative agents were not identified.
- Epidemiologic Evidence: (1) high-risk occupations (farmers, livestock farm workers, butchers, veterinarians, and laboratory personnel), (2) A history of keeping livestock and pet’s animals in recent months, or (3) residency in rural regions and/or living in close proximity (less than 1 km) to livestock farms (cattle, sheep and goats).

Suspected patients were examined by clinical practitioners, and all the symptoms were diagnosed by them. The clinical symptoms and epidemiological evidences were recorded by practitioners in the questionnaires. Eligible individuals were selected by the practitioners and enrolled to the study based on the inclusion criteria. Demographic characteristics, clinical signs and risk factors were recorded for each participant by a standardized questionnaire developed for this study (**S1 Questionnaire**). Blood sample was taken from each patient. Sera were subsequently extracted and used for molecular investigation.

### Ethics statement

This study was approved by the Ethics Committee for Biomedical Research of Tarbiat Modarres University (Ethic Code: IR.TMU.REC.1395.510). The Ethics Committee for Biomedical Research of Tarbiat Modarres University approved the consent procedure, the proposal and protocol of this study, covering all the samples taken (blood), questionnaire and verbal or written informed consent. All participants signed an informed consent: Written informed consent was obtained from adult’s patients and parents of patients below the age of 18. Also, for participants who were illiterate, the consent form was read aloud to them and the interviewer signed the consent form with the permission of these individuals on their behalf.

### *C*. *burnetii* Detection

A 200 μL aliquot of each serum was used for DNA extraction. Genomic DNA was isolated using the Roche High Pure PCR Template Preparation Kit (Roche, Germany), according to the manufacturer's instruction.

All samples were tested by real-time PCR for detection of IS1111 gene of C. *burnetii*, and positive samples were confirmed with nested PCR (**Table 1**). Real-time PCR was performed using specific primers and probe sequences targeting IS1111 gene (**Table 1**). Real-time PCR reactions were performed using the following reaction mixture: 10 μL of 2x RealQ Plus Master Mix for Probe (Ampliqon, Denmark), 900 nM forward primer, 900 nM reverse primer, 200 nM probe and 4 μL of DNA template. Real-time PCR was performed on the Corbett 6000 Rotor-Gene system (Corbett, Victoria, Australia), with a final volume of 20 μL for each reaction. The PCR amplification program were 10 mins at 95°C, followed by 45 cycles of 15 s at 94°C and 60 s at 60°C [14].

**Table 1:** Primer sequences for diagnosis of *C*. *burnetii* IS1111 gene by nested PCR and real-time PCR.

Protocol	Primer Name	Sequence (5→3)	Amplicon size (bp)
**Trans-PCR**	**Trans1**	**TATGTATCCACCGTAGCCAGTC**	687
	**Trans2**	**CCCAACAACACCTCCTTATTC**	
**Nested PCR**	**261F**	**GAGCGAACCATTGGTATCG**	203
	**463R**	**CTTTAACAGCGCTTGAACGT**	
**Real-Time PCR**	**tmQ-koorts4-fw**	**AAAACGGATAAAAAGAGTCTGTGGTT**	70
	**tmQ-koorts4-rv**	**CCACACAAGCGCGATTCAT**	
	**tmQ-koorts4-fam-tamra**	**6-FAM-AAAGCACTCATTGAGCGCCGCG-TAMRA**	

Nested PCR method was performed via two runs of PCR using two sets of primers including Trans1 and Trans2 for first amplification followed by 261F and 463R for second amplification reaction. The products of first PCR were separately used as DNA template in a second round of PCR. Each PCR reaction contained 5μL of DNA, 12.5μL Taq DNA Polymerase Master Mix RED (Ampliqon, Denmark), and 10 pmol/μL from each primer in a final volume of 25μL. PCR was performed in a thermal cycler (Bioneer, South Korea). The first amplification of PCR was done at 95°C for 2 min, followed by five cycles at 94°C for 30 s, 66 to 61°C (touchdown assay) for 1 min and 72°C for 1 min. These cycles were followed by 35 cycles consisting of 94°C for 30 s, 61°C for 30 s, and 72°C for 1 min, then a final extension step of 10 min at 72°C. In the second amplification, the cycling conditions included an initial denaturation of DNA at 94°C for 3 min, followed by 35 cycles at 94°C for 30 s, 50°C for 45 s, 72°C for 1 min, then a final extension step of 10 min at 72°C. The amplicons were electrophoresed on 1.5% agarose gel and visualized under UV light [10].

## Results

In total, 8500 patients were invited to participate in the study and were screened by the clinical practitioners; among them 235 patients had the clinical and epidemiological sings to be suspected for Acute Q fever as the discussed inclusion criteria. Participants who matched the inclusion criteria were selected randomly. Finally, 216 out of 235 suspected febrile patients were enrolled (138 patients from Tabriz County in East Azerbaijan Province and 78 patients from Ghaemshahr County in Mazandaran Province) (**S1 Fig**). The age of participants ranged between 2–82 years with a mean age of 41.5. In total, 61.1% of individuals were male and 38.9% were female. Residency in rural and urban regions among participants was 60.1% and 39.9%, respectively. Also, 61.6% of participants had a history of keeping domestic animals (**Table 2**).

**Table 2:** Demographic categorization of participants in this study.

Variable		Total (%) N = 216	Acute Q fever Cases (%) N = 9
Age (years)	Under 15	16 (7.4)	1 (11.1)
	15–30	48 (22.2)	2 (22.2)
	31–45	61 (28.2)	2 (22.2)
	46–60	64 (29.7)	1 (11.1)
	Over 60	27 (12.5)	3 (33.3)
Gender	Female	84 (38.9)	5 (55.5)
	Male	132 (61.1)	4 (45.5)
Location	Rural	130 (60.1)	2 (22.2)
	Urban	86 (39.9)	7 (77.8)
History of domestic animal keeping	No	83 (38.4)	1 (11.1)
	Yes	133 (61.6)	8 (88.9)

Nine patients’ samples (4.2%) were positive for *C*. *burnetii*. All nine sera samples were positive by nested PCR and real-time PCR (**Table 3**). Prevalence of acute Q fever in Tabriz County and Ghaemshahr County were 3.6% and 5.1%, respectively. Weakness and fatigue (100%), headache (88. 9%), and lethargy (66.7%) were the most prevalent clinical symptoms in positive cases (**Table 4**). Seven (77. 8%) of nine identified patients had a history of keeping livestock. Also, Seven (77. 78%) of the nine detected acute Q fever cases were female and five (55.5%) were residents in rural areas. The demographic and epidemiological findings and were not statistically significant risk factors for Q fever infection.

**Table 3:** Demographic, clinical and laboratory details of nine acute Q fever patients determined by molecular methods in the Iran.

Sample no.	Age/Sex	place of residence	Occupation	Clinical Presentation	IS1111 TaqMan real-time PCR	IS1111 Nested PCR
1	11/Male	Rural	Student	Headache, Lethargy, Chest Pain, Weakness and Fatigue, Atypical Pneumonia	Positive	Positive
2	47/Male	Rural	Farmer	Headache, Chest Pain, Cough, Weakness and Fatigue, Myalgia, shortness of Breath, Arthralgia	Positive	Positive
3	20/Male	Rural	Farmer	Headache, Lethargy, Chest Pain, Cough, Weakness and Fatigue, Myalgia, Shortness of Breath, Arthralgia, Atypical Pneumonia	Positive	Positive
4	40/Male	Rural	Farmer	Headache, Chest Pain, Weakness and Fatigue, Arthralgia	Positive	Positive
5	32/Female	City	Housekeeper	Headache, Lethargy, Weakness and Fatigue, Shortness of Breath	Positive	Positive
6	28/Female	Rural	Housekeeper	Headache, Lethargy, Chest Pain, Cough, Weakness and Fatigue, Atypical Pneumonia	Positive	Positive
7	81/Female	City	Housekeeper	Lethargy, Weakness and Fatigue, Myalgia	Positive	Positive
8	74/Female	Rural	Housekeeper	Headache, Lethargy, Weakness and Fatigue, Myalgia, Arthralgia	Positive	Positive
9	68/Female	Rural	Housekeeper	Headache, Weakness and Fatigue, Myalgia, Arthralgia	Positive	Positive

**Table 4:** Descriptive analysis of clinical signs among participants in the study.

Clinical symptoms		Number of patients (% positive cases)
**Headache**	Yes	176 (4.54)
	No	27 (3.7)
**Lethargy**	Yes	111 (5.4)
	No	92 (3.26)
**Chest pain**	Yes	96 (5.21)
	No	107 (3.74)
**Cough**	Yes	73 (4.11)
	No	130 (4.61)
**Weakness and fatigue**	Yes	173 (5.2)
	No	30 (0)
**Diarrhea**	Yes	17 (5.88)
	No	186 (4.3)
**Myalgia**	Yes	158 (3.16)
	No	45 (8.89)
**Pneumonia**	Yes	89 (3.37)
	No	114 (5.26)
**Shortness of breath**	Yes	68 (4.11)
	No	135 (4.44)

## Discussion

This study is the first molecular investigation of human Q fever cases in the north and north-west of Iran. Among 216 investigated febrile patients in this study, 4.2% were confirmed to be infected with *C*. *burnetii*. Based on recent evidence, Q fever shows high prevalence in livestock and milk and also a high seroprevalence in many different human populations in Iran. The seroprevalence of IgG phase I and II antibodies of Q fever in human has been reported to be 19.80% and 32.86%, respectively. Also, the prevalence of *C*. *burnetii* antibodies in goat, sheep and cattle were reported to be 31.97%, 24.66% and 13.30%, respectively [7]. Despite all evidence, the disease is underestimated by clinicians and the health system in Iran. In fact, most of the clinically diagnosed cases of Q fever have been the outcome of research projects. The results of this study and other reports about human cases in Iran, suggest that the physicians and health care system should pay more attention to diagnosis of Q fever cases in Iran. Special training should be provided for diagnosis of Q fever for clinicians and infectious diseases specialists. In addition to these measures, laboratory diagnostic facilities for the diagnosis of *C*. *burnetii* infection should be expanded throughout the country. It is essential that the healthcare system provides the necessary training for people to understand the disease and to prevent it. Patients with suspected clinical symptoms of acute Q fever must be advised to follow up on specific tests as well as on the completion of appropriate treatment. This way, a higher number of suspected Q fever patients will be diagnosed and treated and thus prevent possible progression of the disease toward chronic Q fever. It is noteworthy that all patients in our study diagnosed with acute Q fever were treated with appropriate antibiotics (Doxycycline and Hydroxychloroquine) and all effectively recovered.

In this study, 4.2% of the 216 suspected febrile patients were positive for IS1111 gene of *C*. *burnetii* as confirmed by nested PCR and real-time PCR. Prevalence of acute Q fever in Tabriz county (East Azarbaijan province) and Ghaemshahr county (Mazandaran province) were 3.6% and 5.3%, respectively. In a similar study that was conducted in northeastern Iran, 7.4% of 92 patients were positive for *C*. *burnetii*, as confirmed by nested PCR [10]. In similar studies conducted in other countries; molecular prevalence of *C*. *burnetii* in acute febrile patients were 0.4% in Senegal [15], 4.5% in India [16] and 14.1% in Poland [17]. The low molecular prevalence of acute Q fever in febrile cases in our study compared to other studies may be due to a number of factors, such as differences in geographical location and climate. More comprehensive studies in this region and other regions of Iran can be helpful for accurate estimation of the *C*. *burnetii* infection in acute illness.

Acute Q fever generally presents as a flu-like illness with wide range of nonspecific clinical manifestations [4]. Patients with cute Q fever may develop respiratory illness or hepatitis. Pneumonia is an important clinical manifestation of acute Q fever, and *C*. *burnetii* might be an underrecognized cause of community-acquired pneumonia [13, 15]. Based on available information and review of the literature, most clinical data of acute Q fever were obtained from patients with Q fever pneumonia [1, 4, 14, 18–20]. Due to the above reasons, we enrolled acute febrile patients with pneumonia (acute lower respiratory tract infections). For future studies, it is recommended that a wider range of clinical symptoms along with pneumonia and undifferentiated fever be considered in order to cast a wider net for the diagnosis of the clinical cases of acute Q fever.

Serologic tests are known as reference methods for diagnosis of clinical cases of Q fever. The reason for the use of serology as detection method is partially the limitation of culture methods in isolation of *C*. *burnetii* and also the strong immune response to the infection (the antibody produced against the bacterium) in the human body, which is easily detectable by serological tests [13]. Unfortunately, serology has limitations in diagnosis of acute febrile illnesses, because it requires two serum specimens (from the acute phase and the convalescent period) and looks for a fourfold increase in antibody content in paired serum samples. Access to the second serum sample takes time (approximately 4 weeks) [4]. Molecular tests are an attractive alternative; they allow for rapid, one-step, diagnosis of patients with acute Q fever and can be performed at an early stage of the *C*. *burnetii* infection [1, 14]. In our study, we developed a diagnostics assay based on real-time PCR for diagnosis of suspected patients and we used nested PCR for confirmation of positive results by Real time-PCR. All nine positive cases were confirmed with nested PCR. Employing this laboratory diagnostic protocol (real-time PCR) can improve and accelerate primary molecular detection, after which the initial positive results can be confirmed by the nested PCR. It is worth noting that the initial and confirmation tests identify and amplify different regions of the IS1111 gene of *C*. *burnetii*, increasing the fidelity of the detection technique. Based on our results, we recommend that molecular tests be combined with the accepted serological tests to diagnose patients with suspected Q fever in shorter time and at earlier stages of the disease.

One of the limitations of our study was the small number of positive cases, which made us unable to do a proper statistical analysis of risk factors and epidemiologic factors. In addition, more precision in the entry of eligible individuals and those who were more closely related to the criteria for diagnosis of acute Q fever, could provide a more precise prevalence of acute Q fever. The combination of molecular tests with serologic tests (as the gold standard diagnostics method) allows for proper identification of all suspected patients. Another limitation of our study was lack of attention to whether antibiotics against *C*. *burnetii* were administered during the sampling time. Therefore, it is suggested that the mentioned limitations should be considered in subsequent studies.

## Supporting information

S1 Checklist STROBE checklist. (DOCX)

S1 Questionnaire Acute Q fever questionnaire. (PDF)

S1 Fig Flowchart diagram of participants. (TIF)
