# Supplementary material for: Genetic evidence of Coxiella burnetii infection in acute febrile illnesses in Iran
Source: PLoS Negl Trop Dis. 2019 Feb 11;13(2):e0007181. doi: 10.1371/journal.pntd.0007181 (PMC6386404; doi:10.1371/journal.pntd.0007181)
Supplement: S1 Questionnaire — (PDF) [file pntd.0007181.s002.pdf]

Acute Q Fever Questionnaire

Questionnaire Code:	Hospital or Health Center name:	
Name of physician:	Name of the Questioner:	Date:
Patient's name:	Address and contact number:	
Age: …… years	Gender: Male☐ Female☐	Marriage Status: Single☐ Married☐
Location Area: Rural ☐ City☐	Occupation:	

Is the job this person is at high risk for Q fever? Yes ☐ No☐

Note: Professionals who are in contact with animals or their products (veterinarians, dairy farmers, butchers), work in laboratories and ... is a high-risk occupation.

What is the reason for the current referral to the doctor? …………

When did the symptoms begin? Day ..... Month ..... Year .....

Has the patient fever in the last few days? Yes ☐ No☐

Does the patient have fever now? Yes ☐ No☐ If the answer is yes, the fever will be recorded: …… C

Which of the following symptoms does the patient have? (According to patient's report and clinical examination)

Headache ☐ Chills☐ Chest pain ☐ Cough☐ Fatigue and weakness☐

Chest pain☐ Diarrhea☐ Acute infection of the lower respiratory tract ☐ Lethargy☐

Myalgia ☐ Atypical pneumonia☐ Shortness of Breath☐ Arthralgia☐ Hepatitis☐

Do you have a history of keeping domestic animals (including livestock and pets) in the last 2 months?

Yes ☐ No☐

If you have a history of keeping domestic animals, what is the type?

Sheep☐ Goat☐ Cattle☐ Pigeon☐ Dogs☐ Cat☐ Horse☐ Other items (listed) .... ☐

Is your place of residence in areas close to the keeping and breeding of livestock animal? Yes☐ No☐

Do you have a history of raw milk and other non-pasteurized dairy products consumption? Yes☐ No☐

Do you have a recent history of abortion? Yes☐ No☐

Do you have a recent history of tick bites? Yes□ No□
Have you recently contacted a newborn or aborted animal? Yes□ No□
Have you recently received antibiotics? Yes□ No□<br><br>If the answer is positive, type it: ....
Laboratory findings (if available):<br><br>Blood culture: ....<br><br>Biochemicals: ....<br><br>Hematology: ....<br><br>Radiography: ....
Study data is given to the all individuals. Does the patient consent to participate in the study?<br><br>Yes□ No□ If the answer is Yes, the patient must sign the consent form.
