# Supplementary figures and images for: Genetic evidence of Coxiella burnetii infection in acute febrile illnesses in Iran
Source: PLoS Negl Trop Dis. 2019 Feb 11;13(2):e0007181. doi: 10.1371/journal.pntd.0007181 (PMC6386404; doi:10.1371/journal.pntd.0007181)

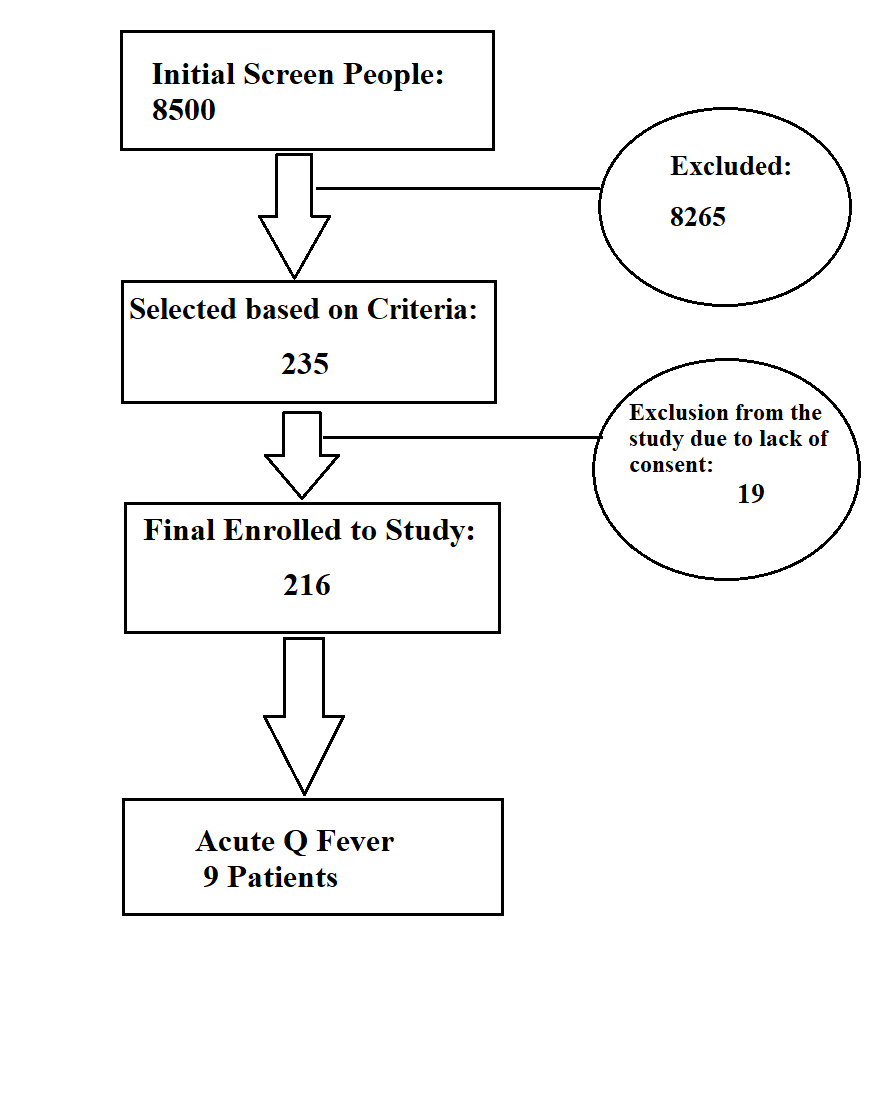

Supplement: S1 Fig — (TIF) [file pntd.0007181.s003.tif]
